# Leber Hereditary Optic Neuropathy: Support, Genetic Prediction and Accurate Genetic Counselling Enhance Family Planning Choices

**DOI:** 10.1111/ceo.14493

**Published:** 2025-02-02

**Authors:** Lisa S. Kearns, Sandra E. Staffieri, David A. Mackey

**Affiliations:** ^1^ Centre for Eye Research Australia Royal Victorian Eye and Ear Hospital East Melbourne Victoria Australia; ^2^ Ophthalmology, Department of Surgery University of Melbourne Parkville Victoria Australia; ^3^ Centre for Ophthalmology and Visual Science, Lions Eye Institute University of Western Australia Nedlands Western Australia Australia; ^4^ School of Medicine, Menzies Institute for Medical Research University of Tasmania Hobart Tasmania Australia

**Keywords:** counselling, genetics, Leber hereditary optic neuropathy, mitochondrial disease

## Abstract

With the increased availability of genetic testing and the addition of mitochondrial genetic variants on disease panels, accurate genetic counselling for individuals and families affected by, or at risk of, Leber hereditary optic neuropathy (LHON) is becoming increasingly relevant. Challenges in providing genetic counselling for LHON include its mitochondrial inheritance pattern, different haplogroups, incomplete penetrance and that it predominantly affects males. Accurate genetic counselling aims to avoid incorrect disease‐risk assessment and delays in either diagnosis or implementation of psychosocial support. Families are also empowered to make autonomous health decisions regarding potential trigger factors for LHON vision loss and informed reproductive choices. Using clinical vignettes, this review demonstrates that an increased awareness of LHON amongst eye care, general and genetic health professionals can address challenges and misconceptions.

## 1 Introduction

Leber hereditary optic neuropathy (LHON) is characterised by an enlarging central or centrocecal scotoma, rendering the individual legally blind [1]. Currently, there is no cure for LHON; however, therapeutic approaches including idebenone, gene therapy and other antioxidants have been reviewed [2, 3]. Management is largely supportive, focusing on improving quality of life, maximising residual vision and the importance of accurate genetic counselling [3].

Earlier epidemiological studies suggested a prevalence of LHON between 1 in 31 000 to 1 in 68 000 [4, 5, 6, 7]. Extending their Australian LHON epidemiology research [8], Mackey et al. more recently investigated the prevalence of LHON–associated variants in two other populations, namely: (1) the UK Biobank [9] and (2) the QSkin study in Australia [10], reporting that 1 in 1000 people carry one of the LHON mtDNA genetic variants [11]. Moreover, not all carriers lose vision. The Australian LHON data suggest that, for male carriers, the risk of losing vision in their lifetime is 17.5% (one in six) compared to 5.4% for females (one in 20) [8]. Naturally, at‐risk LHON families experience high levels of distress and anxiety, pending the uncertainty of blindness. Thus, communication of complex and accurate genetic risk information is of high priority. Genetic counselling forms an integral part of clinical care.

For approximately 95% of people with LHON, the cause is a mitochondrial change at either m.11778G>A (MTND4), m.3460G>A (MTND1) or m.14484T>C (MTDN6) [12]. Other rare LHON variants have been described (http://www.mitomap.org/MITOMAP).

Mitochondrial DNA varies between different populations, forming different haplogroups. These haplogroups are groups of related mitochondrial DNA sequences that share a common ancestor. Some haplogroups may potentially increase the risk of developing LHON–related vision loss. Hudson et al. [13] studied 3613 people from 159 LHON–affected pedigrees and showed the penetrance of:

– m.14484T>C increased on a haplogroup J background, particularly J1
– m.11778G>A increased on haplogroup J background, particularly J2
– m.11778G>A reduced on haplogroup H background
– m.3460G>A increased on haplogroup K background

In further work, Mackey et al. [11] reported mitochondrial haplogroup to be a major factor influencing the risk of LHON–related vision loss. They showed that, of the 1 in 1000 people who had a LHON genetic variant, most were from m.14484T>C pedigrees on low‐risk mitochondrial haplogroup U subclade backgrounds. Thus, by integrating haplotype information with other genetic findings, counselling can be more personalised and provide more accurate guidance for individuals and their families.

Traditionally, LHON has been known as a young man's disease; however, a small number of women, older adults and younger children are also at risk of vision loss [8, 14]. The variable penetrance between families and within branches of the same family implies genetic and/or environmental factors may play a role [15, 16]. The Australian LHON familial epidemiological data also reported a higher incidence of vision loss in children of affected mothers, as well as in children of unaffected women with at least one affected brother [8].

One of the most challenging issues for LHON is counselling ‘at‐risk’ carriers. Although we cannot predict those people ‘at risk’ who will lose vision, it has been suggested that people at risk of LHON vision loss should avoid potential environmental triggers such as smoking, vaping, excess alcohol intake, organic solvents and some medications [15, 16]. When prescribing any medication, clinicians must balance any potential benefit against possible harm. The medications listed below may have a negative effect on mitochondrial function, and their use in individuals affected by LHON or who carry the LHON genetic risk may warrant caution [16, 17, 18].

1. Antimicrobials
  ○ Tetracyclines, aminoglycosides, linezolid, erythromycin, chloramphenicol and ethambutol
2. Hyperbaric oxygen
3. Quaternary ammonium compounds (QACs)
4. Ringer's lactate
5. Antiretrovirals
6. Succinate dehydrogenase (SDH) inhibitors (SDHIs)

A patient‐friendly list can be found online via the website LHON101 available at www.lhon.org/lhon‐101.

To this end, LHON carriers are also encouraged to maintain a healthy diet and use protective head and eye equipment during sport to minimise the risk of blunt head trauma.

LHON, in particular LHON m.3460G>A, has been reported as part of a multisystem condition [19], including Wolff–Parkinson–White (WPW) syndrome [20].

In a small number of LHON families, individuals show LHON‐plus features and MRI scans can show multiple sclerosis (MS)–like features known as Harding's disease, originally described by Harding et al. [21] In addition, and although rare, there are also published reports of ‘double LHON’ variants causing LHON and LHON‐plus [22, 23].

Alorainy et al. [24] published a review of LHON and associations with Harding's disease. They found 88 cases of Harding's disease reported in the literature, with women accounting for the majority of cases (62, or 70.4%), and established a female:male ratio of 2.38:1. In the review, the authors reported the m.11778G>A variant occurred most frequently, accounting for 61 out of 88 cases (69.3%), followed by the m.14484T>C and m.3460G>A variants, at rates of 12.5% and 10.2%, respectively.

Screening for LHON in individuals with MS has been suggested. In a systematic review and meta‐analysis, Pfeffer et al. [25] found that a total of 1666 patients with MS had been screened for LHON variants, and five patients were identified. Although the diagnostic yield was low, detecting a LHON variant is clinically and, from a genetic counselling perspective, important for at‐risk individuals within the family, particularly for women planning a family. Joshi and Kermode [26] reported two siblings, both with m.11778G>A, who manifested different clinical phenotypes: a male with a classic LHON phenotype and a female with an MS‐like illness. Thus, LHON genetic testing could be a consideration in MS patients, particularly those with severe or bilateral visual loss and a matrilineal family history.

The Australian Adaptation of the Mitochondrial Medicine Society recommends individuals with LHON–related vision loss or those at risk undergo periodic neurologic and cardiac evaluations, particularly if there is a family history of other neurological or cardiac problems [27].

Another significant challenge is the timely diagnosis of LHON to facilitate supportive services and any potential treatments or trials. Patients without a known family history have often described a long diagnostic journey. In a qualitative study examining the impact of LHON, the average time from onset of symptoms to final diagnosis was reported as 3–5 months in the United States, 5–7 months in Germany, 3–12 months in France and 1–4 months in the United Kingdom [28]. Increased clinical awareness, optimising referral processes and enhancing diagnostic pathways can improve patient outcomes.

A diagnosis of LHON in a family requires a conversation that clinicians know will profoundly change the lives of all the family members, not just the affected individual. This review will consider actual scenarios from clinical practice that identified challenging situations. Using a clinical vignette format,^1^ we demonstrate that genetic counselling is as critical for family members as for the person who has lost vision.

Clinical Vignette 1: Counselling a person who has recently lost vision.

**FIGURE 1:**
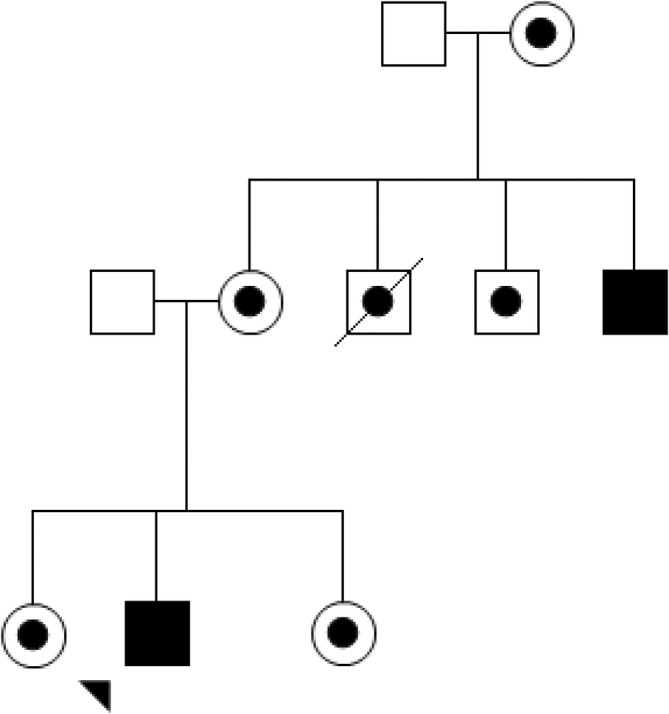
Abridged pedigree from LHONTAS001 Clinical Vignette 1. Jim (denoted by the arrow) is a male aged 18 years with LHON m.11778G>A vision loss. 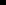, LHON vision loss. 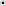, Maternally related at‐risk LHON family members. 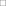, Male (married‐in).

It was just an average day when Jim, then 18 years old (Figure 1), realised he had a major problem with his vision. He could not see the TV screen. He was moving his hands about for perspective and noticed there was an appreciable blind spot obscuring the central vision in his left eye. Following multiple assessments by general practitioners and ophthalmologists, a LHON diagnosis was confirmed. Within 8 months, Jim also lost central vision in his right eye.

As in the case of Clinical Vignette 1, when a person is diagnosed with LHON, individuals or families as a group often seek advice and opinion from specialists experienced with these rare conditions. The family requires clarification regarding prognosis and what ‘going blind’ means. Explaining the extent of vision loss associated with LHON, and that some peripheral vision will be preserved, can reassure the individual that it would be very rare for a person with LHON to have ‘no light perception’ or live in ‘total darkness’. This clarifies what people might imagine by ‘going blind’, reiterating that vision impairment is not binary (blind or not blind) but a spectrum.

## 2 Practical Support

The profound central vision loss makes reading, driving and recognising faces difficult. After potential treatment options have been considered [2, 3] (idebenone, gene therapy and antioxidants) and their evidence‐based merits evaluated, providing the person who has recently lost vision with practical support is essential and perhaps the most beneficial. In Chen et al.'s work [28], all participants agreed on hoping for a cure, but indicated the ideal therapy should focus on autonomy and ensuring their ability to lead a productive vocation and fulfilling social life.

Advances in rehabilitation receive less media coverage than gene therapy, yet many novel technologies, such as driverless cars and optical‐reading technologies, are emerging that may greatly and immediately improve the lives of those with visual impairment. Today, learning to use smartphone applications (apps) and devices can assist people with their everyday tasks, optimise their residual vision and empower them to live a fulfilling and autonomous life.

Siri (Apple) is a well‐known virtual assistant; others include Google Assistant, Amazon Alexa, Cortana and Samsung Bixby. Seeing AI is a freely available smartphone application developed by Microsoft (https://www.microsoft.com/en‐us/ai/seeing‐ai). The app can read text, product bar codes, monetary notes and describe scenery as well as identify and recognise faces. In an online survey by Griffin‐Shirley et al. [29], over 95% of participants rated vision‐assistive apps useful and 91% of people found they were accessible. However, Malkin et al. [30] found that in a cohort of visually impaired people over 50 years of age, although 90% of them had a smartphone, only 6% used vision‐assistive apps; all but one of the participants (98%) were interested in them. The primary reason for limited use of the apps was lack of awareness. With technology continuing to evolve and holding such potential for people who are vision impaired, clinicians need to stay current to ensure their patients/clients can benefit from these advances.

People with LHON may also require assistance with daily activities, education or employment. The visual acuity of most people with LHON–related vision loss is so poor that individuals cannot meet regulated vision standards to drive a car, forklift or other machinery. Often, people require advice on what is possible to continue to do safely and what help can be provided. Low‐vision support organisations can facilitate guidance on adaptation and retraining to re‐enter the workforce.

## 3 Psychosocial Support

Sudden loss of vision is a life‐changing experience. Clinically, people with LHON face similar challenges to those with Stargardt disease, an inherited retinal disease (IRD) also characterised by central vision loss. Using grounded theory, Bryan, LeRoy, and Lu [31] analysed the written essays of participants with Stargardt disease. Common themes were that affected individuals often felt a loss of independence after losing their driver's licence, were distressed about not recognising friends or family and were frustrated by being unable to read signs and requiring assistance navigating unfamiliar locations. Participants in the study also expressed fears about how the disease would affect their personal and professional lives.

In a series of semi‐structured interviews with both affected individuals and caregivers [32], LHON was described as a disabling condition disrupting both current and future plans. It affected all key dimensions of participants' lives: mood, physical capabilities, relationships, work/studies, finances and recreational activities. As a result, participants felt frustrated and heavily dependent on caregivers. People with LHON–related vision loss reported that going from being fully sighted to living with LHON required resilience, pragmatism and determination to develop the practical and emotional coping skills to manage their environments and progress with their lives [33].

Chen et al. [28] also published a qualitative study including 17 people with LHON m.11778G>A vision loss and 17 relatives. The researchers explored the impact of LHON on the patient and their relatives' lives at the time of, and following, diagnosis. Similar to earlier studies, loss of independence and reliance on family and friends were frequent themes identified in the study. Financial impacts included the purchase of medications and visual aids, particularly for participants in countries where idebenone or visual aids were not fully subsidised or reimbursed. Similarly, for a cohort of LHON patients and caregivers in Czechia and Slovakia, the direct non‐medical and indirect costs of LHON demonstrated that families face a socioeconomic burden [34].

A LHON diagnosis has been demonstrated to have a negative impact on quality of life [35]. Garcia et al. [36] reported nearly half (49.5%) of participants who had profound vision loss from LHON met the criteria for major depressive disorder from the Diagnostic and Statistical Manual of Mental Disorders‐V, with older adults having lower psychological well‐being than younger participants [37].

Psychological support for any type of vision loss is important for coping and adaptation. Some people may require additional referrals for grief counselling and/or a mental health plan to help support them during challenging times. Further research into the support needs of LHON families could help understand enablers and barriers in coping with LHON vision loss, supporting a family member or living with uncertainty.

In some instances, people affected by LHON and/or family members may choose to engage with support groups, sometimes at the time of diagnosis or in the years that follow. Hearing about success, achievements and strategies for coping with LHON can be helpful. Such groups include the not‐for‐profit foundations for mitochondrial diseases, LHON–specific Facebook groups and other vision‐rehabilitation support groups. Support groups also play a crucial role in creating a bridge between researchers and families affected by LHON. Information exchange between clinicians and researchers ensures that patients receive accurate, evidence‐based updates on the latest information and clinical trials and, in turn, clinicians and researchers incorporate the lived experiences and knowledge of those affected by the condition in their management approaches.

Written vision reports are a key component of the documentation needed to access vision rehabilitation and supportive services, for example, the National Disability Insurance Scheme in Australia. Eye care providers must provide reports that specify diagnosis, visual acuity and visual fields, and also demonstrate the functional impact on the individual (practical, emotional, educational and occupational challenges), specify that vision loss is typically permanent, with no cure, and recommend any available assistance devices/services to substantiate the applicant's claim for assistance. For example, a template for Ophthalmologists or eye care providers specifically around Leber Hereditary Optic Neuropathy (LHON) and functional impact is available at https://www.mito.org.au/resource/fact‐sheet‐page/ndis‐assistance‐for‐lhon/. In Australia, individuals affected by LHON whose best‐corrected visual acuity is less than or equal to 6/60 (Snellen chart) in each eye qualify for the disability support pension (DSP). Application forms are available from Services Australia (formerly Centrelink) and supporting medical evidence is required. Prompt and accurate completion of documents can mitigate rejected applications, which otherwise result in significant distress, compounded by challenges of managing severe vision loss and uncertainty for the future.

Clinical Vignette 2: Incorrect counselling for unaffected/non‐carrier female

**FIGURE 2:**
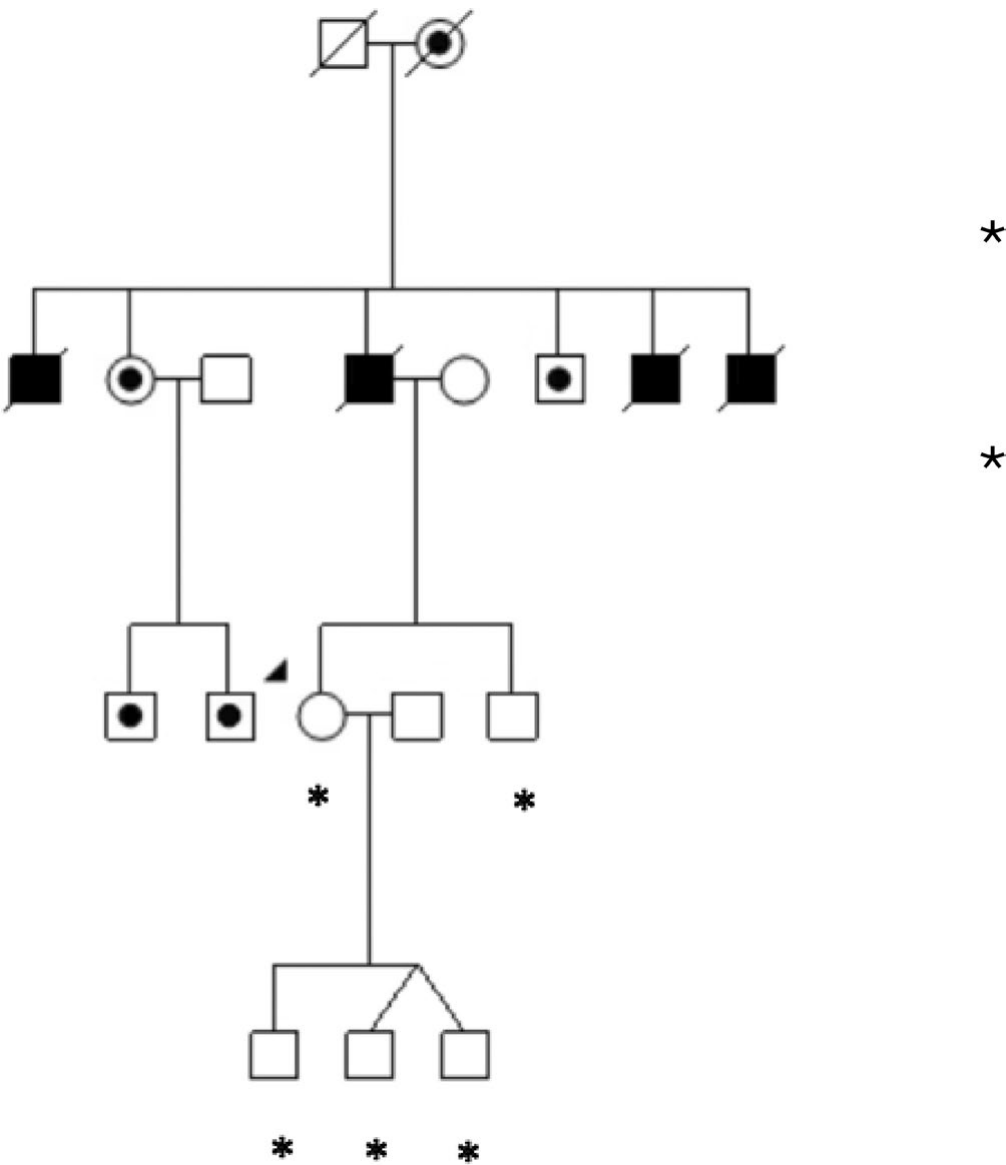
Abridged pedigree from LHONTAS001. Clinical Vignette 2. Unaffected/non‐carrier woman (Alice) denoted by the arrow. 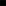, LHON vision loss. 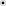, Maternally related at‐risk LHON family members. 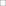, Male (married‐in). 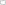, Female (married‐in). 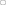, * Female non‐LHON carrier. 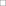, * Male non‐LHON carrier.

Alice took her three sons [5‐year‐old twins and 3‐year‐old singleton] (Figure 2) for a routine eye assessment and was informed by her eye care provider that the boys could inherit the LHON genetic risk and may develop vision loss as severe as their maternal grandfather's (Figure 2). The affected man (Alice's father) was known to have the m.11778G>A, lost vision as a teenager and belonged to one of the largest LHON Australian pedigrees, LHONTAS001.

Clinical Vignette 2 demonstrates the perils of inaccurate risk assessment for families. Alice faced a significant psychosocial burden with the familial implications for her sons, but also her own uncertain personal risk of vision loss. A male affected with LHON *cannot* pass on the specific LHON genetic risk. Therefore, in this case, Alice was provided incorrect information and lived with the unnecessary emotional distress of potential future vision loss for herself and her children.

Although Alice could be reassured she was not a carrier of the familial LHON genetic risk, her paternal aunt as an obligate carrier would not only be at risk of vision loss herself but could also pass on the LHON genetic risk to her children and she should receive counselling.

Accurate LHON risk information is essential for families, not only at the time of initial diagnosis for the person who has lost vision but also for family members, with careful consideration at the different stages of the life cycle, that is, family planning or exploring risk for children. Individuals and families often discuss their concerns with an eye care provider, thus an increased awareness amongst our colleagues can help mitigate the impact of incorrect counselling.

Ophthalmic genetics is an ever‐evolving field and there is an increased need to support and enhance genetic education for eye care providers. In the context of IRD, Britten‐Jones et al. [38] surveyed Australian and New Zealand optometrists' attitudes and perceptions, and knowledge about genetic testing and gene therapy for IRD. Amongst their study respondents, knowledge regarding ocular genetics and gene therapy was modest amongst optometrists, with a median knowledge score of six out of nine. Conversely, an earlier study from 2015 by Ganne, Garrioch, and Votruba [39] found low genetic literacy amongst 35 UK–based optometrists, dispensing optometrists and optometry clinic staff.

There is limited research regarding genetic knowledge or awareness amongst eye care professionals specifically for LHON or other mitochondrially inherited conditions. A study presented at the 2023 Association for Research in Vision Ophthalmology annual congress by Trier et al. [40] showed both ophthalmologists and neurologists had increased competence and confidence in LHON assessment and management following the online medical information. The material also increased their knowledge about current and emerging therapies [40].

Given LHON can be more complex with regards to mitochondrial inheritance, haplogroup, and incomplete penetrance compared to other Mendelian inherited eye conditions such as IRDs, further education for eye health professionals is warranted.

Clinical Vignette 3: Incorrect counselling for a female, now affected with LHON vision loss.

**FIGURE 3:**
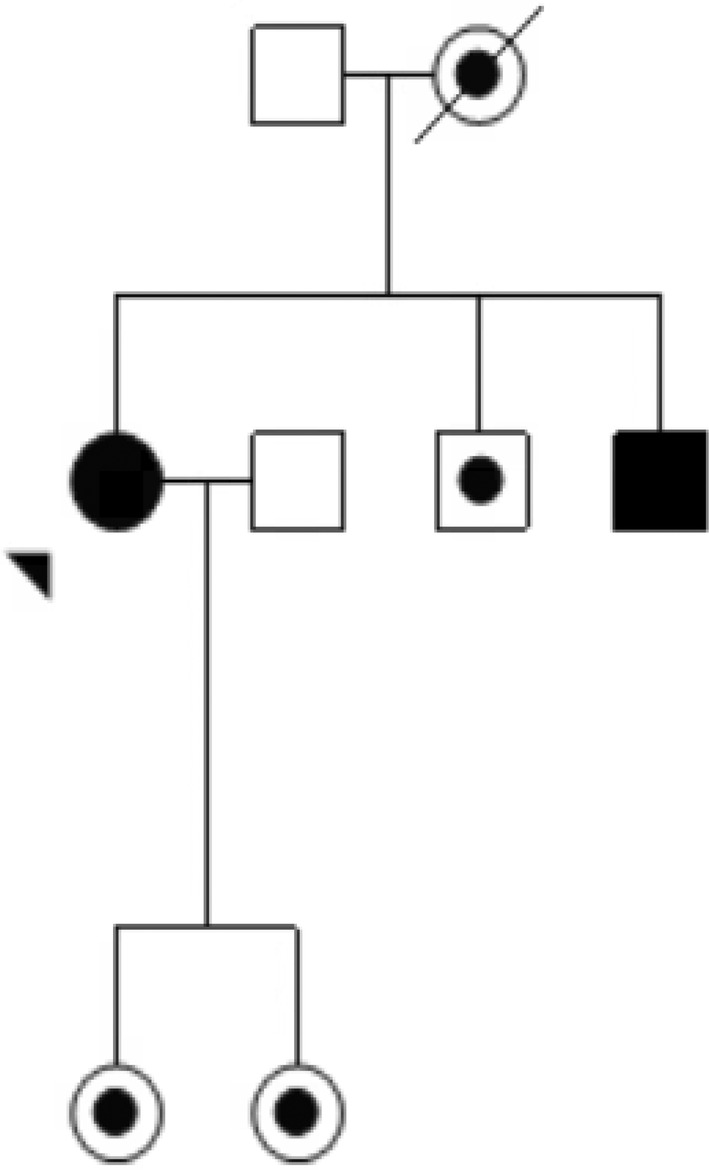
Abridged pedigree from LHONTAS001. Clinical Vignette 3. Cindy (denoted by the arrow) is a female in her 50s with LHON m.11778G>A vision loss. 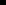, LHON vision loss. 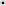, Maternally related at‐risk LHON family members. 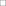, Male (married‐in).

Cindy, in her mid‐50s and postmenopausal, developed vision loss initially in her left eye, followed soon after by vision loss in her right eye (Figure 3). She had two brothers, the younger of whom lost vision at age 15 years from LHON m.11778G>A. The advice she had received at the time of her brother's diagnosis was that only males can be affected by LHON. When she experienced severe vision loss in her sixth decade, her family history of LHON was not considered relevant and Cindy's reported time to diagnosis was long. This diagnosis was complicated largely due to clinicians interpreting LHON as a male‐only condition, and thus her vision loss was a diagnostic dilemma.

Our third vignette highlights an atypical pedigree and diagnostic dilemma not unique to reports in the scientific literature [41, 42]. Although epidemiology studies by both Lopez Sanchez et al. [8] and Poincenot, Pearson, and Karanjia [14] confirmed a male preponderance in LHON, a small number of women were also affected. In 1990, Franks and Sanders [43] described four women from three different families who developed bilateral, severe optic nerve disease. Two of the women described in the article were mislabelled with a diagnosis of MS–related disease, and LHON was only a consideration when a male family member subsequently lost vision [43]. It has been noted that women often experience LHON–related vision loss at an older age compared to men. In 1999, Leo‐Cottler and Christ‐Adler [44] reported a cohort of 11 women with LHON–related vision loss compared to 66 adult males and four male children. In the cohort [44], women were older than men at the onset of disease (19–55 years, average 31.3 years; 15–53 years, average 24.3 years, respectively), suggesting age differences. More recently, a Japanese study also confirmed that the age of onset was higher in women than in men, with the median 31 years (interquartile range 17–45.5 years) in males and 49.5 years (interquartile range 38.8–54.0 years) in females [7].

The higher prevalence of LHON in males compared to females suggests sex hormones may play a role. It is postulated that oestrogen may have a protective effect for retinal ganglion cells and other nerve cells [45], and this may delay the onset of vision loss in females until menopause, when oestrogen levels decrease.

Although other causes of vision loss should be appropriately explored, LHON should always be a consideration in an adult woman with unexplained vision loss. First, timely diagnosis is essential, as most treatments for LHON will largely be most beneficial when initiated early in the disease course. Second, unlike affected men, women are at risk of having current and/or future children affected.

Genetic counselling is highly beneficial to affected women in LHON pedigrees (affected, unaffected and mothers of children with LHON) to help them understand the genetics, inheritance pattern and their personal risk of vision loss. The discovery of a LHON genetic variant may result in mothers experiencing a burden of guilt, and they will often express concern that their own children could also develop LHON vision loss. ^28^

Clinical Vignette 4: Diagnosis overlooked because of older age at presentation.

**FIGURE 4:**
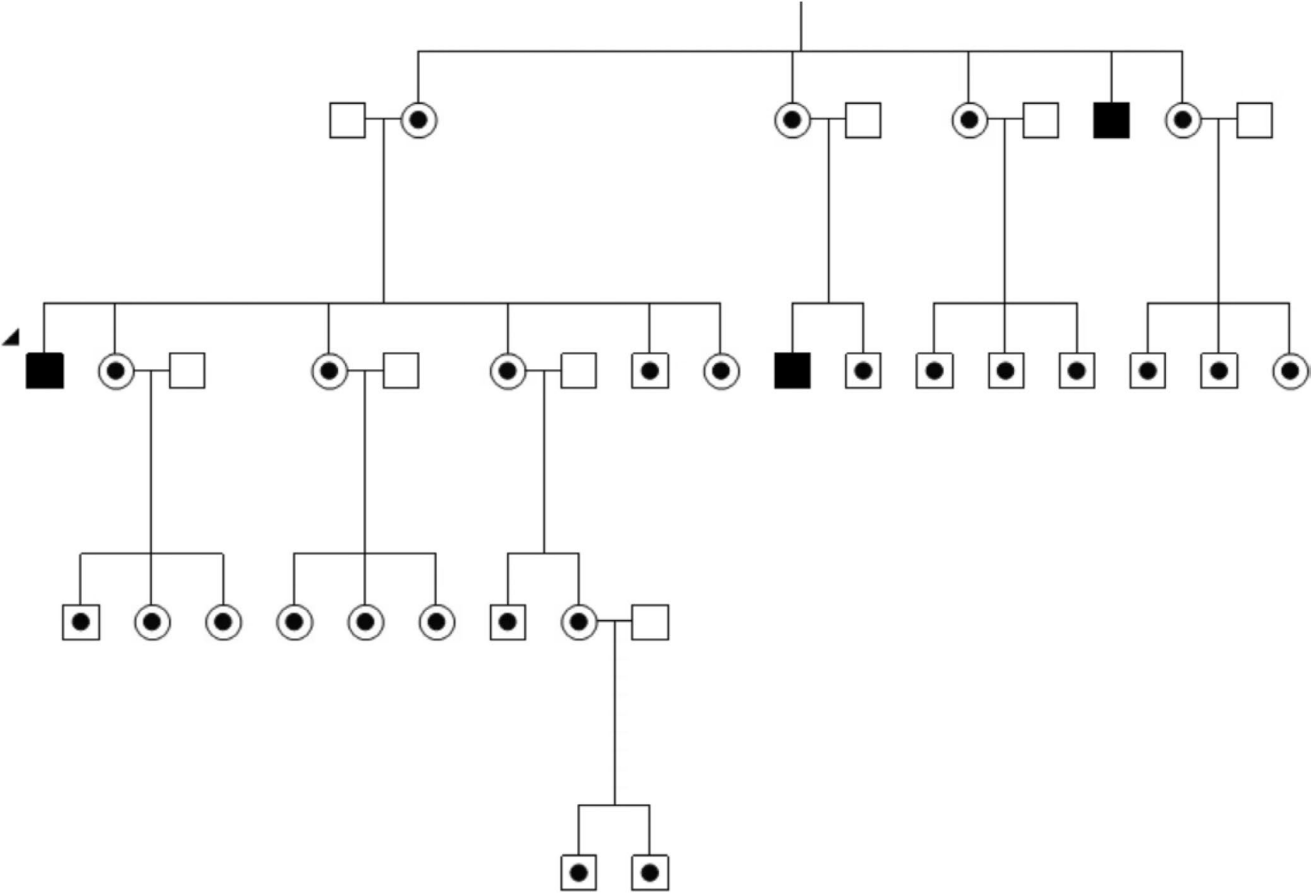
Abridged pedigree from LHONTAS002. Thomas (denoted by the arrow) is a male, 50 years old with LHON m.14484T>C vision loss. 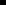, LHON vision loss. 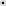, Maternally related at‐risk LHON family members. 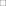, Male (married‐in).

Thomas, a 50‐year‐old male, presented with sudden loss of vision in his right eye, followed by the left eye over the following weeks (Figure 4). There was a family history of LHON, with a maternal uncle and a male cousin losing vision from LHON attributed to m.11778G>A. Despite the clinical features of the optic neuropathy, Thomas was initially presumed to be too old for a LHON diagnosis.

Our fourth vignette dispels the myth that LHON is a young man's disease. It reinforces that LHON should be considered a differential diagnosis in sudden, unexplained vision loss in mature or older adults.

Dimitriadis et al. [46] described a cohort of 20 participants aged greater than 50 years (15 males and 5 females) with LHON–related vision loss and 89 asymptomatic carriers in the same age bracket. The study demonstrated that those who had LHON vision loss also had a significantly higher mean cumulative tobacco and alcohol consumption compared to unaffected carriers.

Increased alcohol consumption and smoking during the COVID‐19 pandemic were also thought to be a contributing factor in the presentation of LHON cases in older people [47]. However, late‐onset LHON vision loss has also been described in people not thought to have cumulative trigger factors of smoking and alcohol [48].

Layrolle et al. [16] recently published an updated review on the environmental triggers, other genetic factors (including haplotype data), lifestyle, dietary supplements, and common chemicals and drugs that may play a role in LHON expression. In the context of increased awareness of LHON, families should be provided recommendations on potential triggers to reduce the risk of LHON–associated vision loss.

Clinical Vignette 5: Childhood‐onset LHON

**FIGURE 5:**
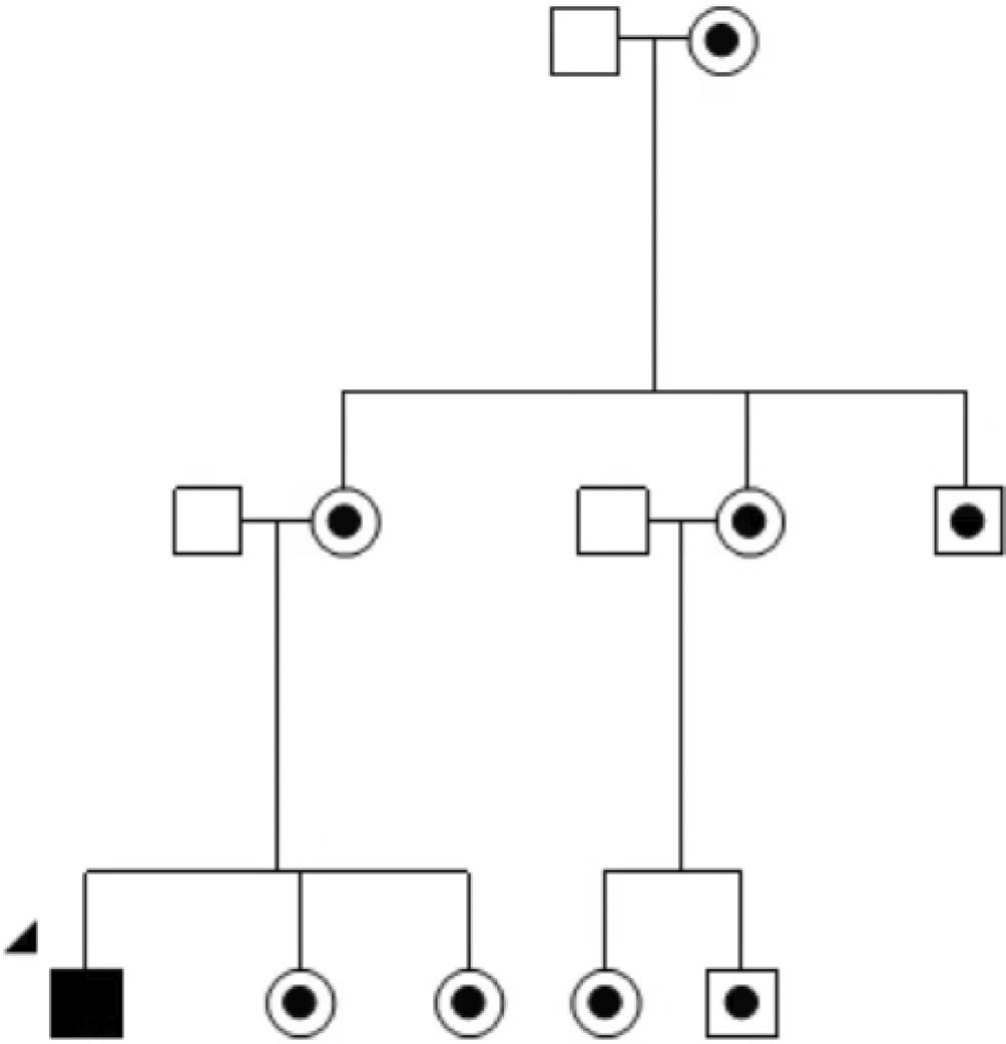
Pedigree from LHONVIC043. Clinical Vignette 5. Sam (denoted by the arrow) is a 10‐year‐old male with LHON (1778mtDNA) vision loss. 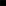, LHON vision loss. 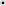, Maternally related at‐risk LHON family members. 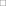, Male (married‐in).

Sam, a 10‐year‐old male, presented with unexplained sudden vision loss in his right eye (Figure 5). He was already under the care of an eye care provider for anisometropic amblyopia in his left eye. His family history for eye disease was unremarkable, and he was promptly referred for electrophysiology testing. Visual evoked potential (VEPs) and other investigations were suggestive of bilateral optic neuropathy, and genetic testing for LHON genetic variants was requested. At this point, the vision in the better‐seeing right eye had also significantly decreased. Genetic testing identified a LHON m.11778G>A genetic variant. The family were surprised and understandably devastated by the news.

LHON–related vision loss before the age of 12 is often referred to as childhood‐onset LHON [49, 50, 51]. It has also been reported that children who lose vision from LHON before the age of 9 years have a better visual prognosis compared with those who experience vision changes in later years [51].

Although rare, Clinical Vignette 5 demonstrates that clinicians should consider the possibility of LHON in childhood against the background of unexplained subnormal vision.

Petrovic Pajic et al. [52] presented a case series of three adult patients with genetically confirmed LHON. Each patient had one eye that was amblyopic from childhood, and they then presented with LHON in the better eye later in life. Strabismic amblyopia was thought to explain the reduced vision of two of the three patients, and the other patient was initially diagnosed as having hyperopic amblyopia. Petrovic Pajic postulated whether the low vision in the first eye in childhood was due to an early LHON episode rather than true amblyopia. Their study demonstrated that careful and thorough ophthalmic testing, which includes examination of the optic nerve head with OCT, visual fields and colour vision, is valuable in any child with amblyopia not responding to conventional treatment.

Research has suggested the impact of a disability in a child extends beyond the child. Data from a longitudinal Australian study of 4935 children and parents, published by Chen et al. [53] revealed that after adjusting for demographic and socioeconomic factors, parents of children with disabilities were approximately 2.10 times (*p* < 0.001, 95% CI: 1.82, 2.42) more likely to experience mental health problems compared to parents of children without disabilities. It was also reported that mental health concerns for parents of children with disabilities increased as children grew older (4–5 years = 28.57% and 16–17 years = 44.92%), in comparison to parents of children without disabilities (4–5 years = 19.61% and 16–17 years = 25.83%).

Little research has been done on parental experiences following diagnosis and caring for a child with sudden vision loss from LHON, particularly in pedigrees with high penetrance and neurological associations. However, it has been reported that parents of a child with a rare disease often face challenges managing their child's needs and are frustrated by health professionals' lack of knowledge and awareness of the condition [54, 55].

Genetic counselling can facilitate positive and informed discussions between mothers and their children about the inheritance pattern in LHON. This will be more significant as the children approach reproductive age and are planning their own family. In some instances, counselling, as well as participating in support groups or talking with mothers who have a child with LHON vision loss, can also help alleviate and normalise feelings of guilt.

Both genetic and psychological counselling can also help provide hope for parents, showing them that with additional support, they can still hold on to future dreams for their child. Hope may have a positive impact on patient outcomes, such as quality of life, and helps people find inner strength, cope with suffering and accept their situation [56]. However, as described by Koenig Kellas et al. [57], supporting hope in a loved one is difficult for relatives and carers, particularly when trying to strike a balance between honesty and hope.

Research suggests uncertainty can negatively affect parental coping and adaptation. Living with the genetic risk of blindness and uncertainty for other at‐risk children is no different. In a study of uncertainty and hope amongst mothers of children with Duchenne muscular dystrophy, Bell et al. [58] showed that uncertainty and hope are both independent predictors of coping efficacy. Their findings support the possibility that hope can offer a way to manage uncertainty. Although there is no current treatment for LHON, healthcare providers play an important role and can assist hope in low‐hope individuals. Many immediate and extended family members (particularly maternally related) grieve when their loved one has experienced LHON vision loss. It is critical that families have access to appropriate practical care strategies and psychosocial support to help facilitate adaptation.

## 4 Family Planning Considerations

Now a teenager, Sam was 10 years old when he lost his vision. He will not carry the anxiety of passing on the LHON genetic risk, unlike his sisters and female cousins who are approaching reproductive age. Women (either with LHON vision loss or an at‐risk sister) will pass on the LHON genetic risk to all their children and thus reproductive decision‐making can be complex. Genetic counselling provides critical insight into varied reproductive options and enables individuals and couples to make informed decisions that are right for them.

With the LHON genetic risk, families will often carefully consider their personal values, beliefs, advantages and disadvantages of the different reproductive options. Prospective parents with a risk of LHON may consider not having children or adopting or fostering children. Fulfilling their desire to have biologically related children, some may opt for natural conception and accept the genetic risk of LHON.

Worldwide, assisted reproductive technologies (ARTs) including in vitro fertilisation (IVF), are most frequently performed to overcome infertility [59]. The technology is also used for fertility preservation and preimplantation genetic testing (PGT) to avoid passing on a known genetic risk for disease to offspring [59]. For LHON, women who are considering IVF may choose the option of non‐LHON carrier donor eggs or embryos. In this case, the child would not be genetically related to one or both parents. However, often there is a desire to have a genetically related child. This may prompt some families to use IVF and gender selection to select female embryos for implantation given that the risk of LHON–related vision loss in females is lower.

Another genetic technology poised to make a difference to families with LHON, and other mitochondrial inherited conditions, is the use of mitochondrial donation [60]. This IVF–based technique replaces the specific mitochondrial genetic variant in the woman's eggs with healthy mitochondria from a donor egg, reducing the genetic risk of LHON transmission.

Mitochondrial donation requires changes to legislation governing ART and embryo research in many countries [61]. A challenge to mitochondrial donation is the prohibition of germline gene editing in nuclear DNA, but also mitochondrial DNA [62].

In the United Kingdom, the Human Fertilisation and Embryology Authority (HFEA) regulates human fertility treatment and scientific research involving human embryos. In 2015, they were the first in the world to establish a regulated system for mitochondrial donation [60]. Currently, the Newcastle Fertility Clinic (Newcastle Hospitals NHS Foundation Trust) is the only licensed clinic to perform mitochondrial donation. It can only be offered to people for whom there is a risk of passing on a serious mitochondrial disease. Individuals and families apply to the HFEA, and decisions are made on a case‐by‐case basis.

Until recently, legislation prohibited mitochondrial donation in Australia [63]. However, in March 2022, following a series of public consultations, an amendment ‘Maeve's Law’, formally called the Mitochondrial Donation Law Reform Bill 2021, was approved by Parliament to legalise mitochondrial donation for research, training and human reproductive purposes [64]. Mitochondrial donation will initially be offered under a carefully regulated clinical trial framework. The MitoHOPE (Healthy Outcomes Pilot and Evaluation) Program is an introduction of mitochondrial donation and the first of its kind in Australia [65]. Women and families who are undertaking family planning can be referred to a clinical genetic service to discuss available options to allow informed decision‐making.

## 5 Conclusion

Vision loss from any eye condition is devastating at whatever time it occurs in one's life. Eye care providers are often the first port of call for people experiencing eye problems or seeking advice for their inherited eye condition. It is essential eye care providers are aware of LHON presenting in atypical scenarios, that is, in females or onset in childhood or late adulthood. In addition, they must ensure ophthalmic reports are sufficiently detailed to include the functional impact of LHON so that supportive agencies and services can promptly action a management package for the affected individual. LHON may deeply affect many individuals in the nuclear family and beyond, thus accurate risk information is essential, particularly for family planning and ensuring families can make informed choices. Given the limited research in the area, understanding the perspective of either living with LHON or the threat of impending blindness, barriers or enablers to adjusting to new vision loss, and the gaps in current support needs is required to optimise quality of life for individuals and families.

## Conflicts of Interest

The authors declare no conflicts of interest.

## Data Availability

The data that support the findings of this study are available from the corresponding author upon reasonable request.

## Endnotes

^1^
All clinical pedigrees are used with patient permission, pseudonyms are used and data are anonymised to protect patient privacy.
